# Use of *Parthenium hysterophorus* with synthetic chelator for enhanced uptake of cadmium and lead from contaminated soils—a step toward better public health

**DOI:** 10.3389/fpubh.2022.1009479

**Published:** 2022-10-13

**Authors:** Ujala Ejaz, Shujaul Mulk Khan, Muhammad Aqeel, Noreen Khalid, Wajiha Sarfraz, Nayab Naeem, Heesup Han, Jongsik Yu, Gong Yue, António Raposo

**Affiliations:** ^1^Department of Plant Sciences, Quaid-i-Azam University, Islamabad, Pakistan; ^2^Member, Pakistan Academy of Sciences, Islamabad, Pakistan; ^3^State Key Laboratory of Grassland Agro-ecosystems, College of Ecology, Lanzhou University, Lanzhou, China; ^4^Department of Botany, GC Women University, Sialkot, Pakistan; ^5^College of Hospitality and Tourism Management, Sejong University, Seoul, South Korea; ^6^College of Business Division of Tourism and Hotel Management, Cheongju University, Cheongju-si, South Korea; ^7^Business School Tourism and Hospitality Management, Xuzhou University of Technology, Xuzhou City, China; ^8^CBIOS (Research Center for Biosciences and Health Technologies), Universidade Lusófona de Humanidades e Tecnologias, Lisboa, Portugal

**Keywords:** phytoextraction, *Parthenium hysterophorous*, cadmium, lead, EDTA, soil remediation, weeds, soil pollution

## Abstract

*Parthenium hysterophorus* L. is a vigorous plant species with cosmopolitan distribution. It can uptake considerable quantities of heavy metals from the soil and accrue these metals in its different tissue. The use of chelating agent i.e., Ethylenediaminetetraacetic acid (EDTA) can boost up metal uptake capacity. Pot experiment was performed to evaluate phytoextraction potential of *P. hysterophorus* for lead (Pb) and cadmium (Cd) with and without the aid of EDTA chelator. Shoot length, weight of root and shoot (both fresh and dry), leaves number, and chlorophyll contents of *P. hysterophorus* got reduced with an increase in metal uptake. The results revealed the highest concentration of Cd in shoot without and with EDTA was 283.6 and 300.1 mg kg^−1^, correspondingly. Increase in Pb concentration was also boosted up by the EDTA from its maximum concentration in shoot 4.30–9.56 mg kg^−1^. Generally, Pb and Cd concentrations were greater in shoots of *P. hysterophorus* than the roots regardless of EDTA in the treatments. EDTA also impacted positively the accumulation of essential ions K^+^, Na^+^, and Ca^+2^ in *P. hysterophorus*. The capacity of *P. hysterophorus* to accumulate Pb and Cd found to be increased with EDTA in the soil. Bringing metals level in the soil in accordance to the WHO standards can improve the ecosystem as well as public health.

## Introduction

Heavy metals or potentially toxic elements have become a great risk to environmental safety due to continuous agricultural practices, industrial expansion and a rise in the population. Heavy metals do not decompose and as a result, they continue to build up in the environment (1). Application of fertilizers, sewage sludge, urban traffic, chemical emissions from industries and mining processes are the examples of anthropogenic sources (2). These appliances are responsible for increasing heavy metal concentrations in soil (3, 4). Heavy metals in agricultural lands and natural resources may pose a threat to public health due to their potential access to food chains (5–8). Constant application of pesticides and chemical fertilizers may build up the number of heavy metals in the soil (9, 10). Dry and wet waste residues from various point sources such as the steel industry, metal refineries, metal smelters, cement industries, and foundries also increase heavy metals to the soils. But mainly the combustion from engines using lead (Pb) improved petrol is liable for major production of Pb in soils adjacent to roads. According to some recent studies, vehicle exhausts are the biggest sources of Pb and Cd in the environment (11, 12).

Pb is a potentially toxic heavy metal above the permissible limits. Apart from public sewage sludge and leaded gasoline, it also results from mining, oils, paints, and unstable materials. Pb is widely used in many industrial applications as a tetraethyl Pb [(CH_3_CH_2_)_4_ Pb], a longtime motor gasoline ingredient and a current stabilizer to certain petroleum, producing the most common heavy metal contaminants in the soil (13, 14). Increase in lead pollution contributes to soil toxicity and also disturb microbial diversity (15–17). According to the U.S. Environmental Protection Agency, 207,000 Pb-contaminated regions in the U.S. including millions of farms need to be cleaned (18). This dangerous pollutant could be taken up *via* plants and reaches the body by consumption of contaminated plant products and gathered in different organs. In severe cases, it may cause human mortality (19). Cd is an extremely cancer causing material that can trigger dangerous responses even in minute quantities (20). Cd can be taken up by plants and ultimately it gets transferred into the food chain (5). Like Pb, Cd also comes from both manmade and natural resources and has a major impact on the disruption of environmental quality. Road traffic could be a big source of Cd in adjacent areas as Cd on large scale is used in lead-acid batteries (12).

Many remediation techniques have been devised so far to handle the contaminated soils (21–23). In addition to traditional techniques of soil purification; phytoextraction is suitable for severely contaminated sites, while phytostabilization is widely used to remediate slight heavy metal contaminated soils (3, 24, 25). Phytoextraction is one of the phytoremediation type, in which the absorption and collecting of metals occur into plant aerial parts from polluted soil. Using plants that could bear a high level of heavy metals is crucial. Chelating substances have been used in metal contaminated soils as decontaminants to boost up phytoextraction lately (26). The cost of phytoextraction could be more than 10 times lower per hectare than standard soil remediation methods such as thermal soil remediation, air sparging and encapsulation. Phytoextraction can be implemented in all locations where soil and weather are appropriate for plant development (27). However, the capability of a plant to accumulate metals from the soil depends upon plant species and their growth habits. The plants selected for phytoextraction should have a rapid growth rate, more production of bio-mass, hyperaccumulater of heavy metal, broadly spread, stem to shoot translocation, toleration of toxic heavy metal impacts, pathogen and pest resistance, welladapted to environmental circumstances, simple to grow and harvest, and do not attract herbivores to prevent entering the food chain (28, 29).

One such plant is *Parthenium hyterophorus* L. This plant is preferred because of its rapid expansion and inedibility to herbivores. *P. hysterophorus* belongs to family Asteraceae, also recognized as congress grass, is an annual herb, invasive weed found across Pakistan and the world. It has been established to perform a fundamental function in the accretion of toxic metals particularly in contaminated sites (30). *P. hysterophorus* has a very high potential for remediating soils polluted with Cd and Pb (11). Phytoextraction employing *P. hysterophorus* is a cost-effective and possible remedy for the cleanup of Cd and Pb polluted soils. In Pakistan, this weed is scattering in harsh environments, despoiled areas, rocky crevices, across waterways, roads, and railway lines. It has also been recently identified in cultivated land.

While chelating substances are used as decontaminants in polluted soils to smooth the process of phytoextraction. Previous knowledge confirms the use of artificial metal chelates like EDTA in soils improved Pb uptake with the help of plants (31). EDTA produces soluble metal EDTA complexes, due to its strong affinity toward heavy metals, assisting in the solubility of soil metals and therefore improving metal accessibility to plants (32). However, EDTA is generally known for its excellent property of metal absorption in soil, but it is also toxic to some species and inhibits their growth. Additionally, EDTA has a weak biodegradation rate and stays for long time in environment (33). EDTA is so far the most proven and successful chelator for removing hazardous metals from soil (34). Some studies previously used phytoremediation for some other metals with or without using EDTA as a phytochelator, or they used some other plants/microbes as phytoremediators (35–38). It was hypothesized that the EDTA is the most potent chelator in lowering Pb and Cd bioaccessibility in soil (34). Both of these metals as well as *P. hysterophorus* are commonly found in the soils along roads. Keeping this information in mind, we aimed to study the metal uptake capability of this fast-growing weed (*P. hysterophorus*) with the help of EDTA chelator. This study used *P. hysterophorus* for the first time for its phytoremediation capabilities in combination with chelator (EDTA). Our objectives are (1) to use Pb and Cd simultaneously with EDTA to evaluate the phytoextraction potential of *P. hysterophorus*, whether the use of EDTA has any impact in decontaminating these metals by *P. hysterophorus* and (2) assess the effect of these combinations on growth and functioning of the plant.

## Materials and methods

### Experimental design

Clay pots of 30 cm in diameter were used in this Completely Randomized Designed (CRD) experiment. A total of 30 pots, filled with soil from Botanical Garden of GCWUS, were used in two sets of treatments assigning 15 pots for each treatment set. Ten kilograms of pure and dried sandy loam soil was filled up in each experimental pot. Seeds of *P. hysterophorous* were gathered from plants growing in non-contaminated areas and each container had ten seeds. *P. hysterophorous* doesn't require a lot of water to flourish due to its wild nature thus, tap water is provided twice a week in accordance with the plant's requirements. The temperature at the time of seed sowing was 36/20°C (day/night) and 24/12°C at the harvesting time period. After plant germination 2 sets of treatments each with 3 replicates were applied to the soils in the pots in 2 weeks (Table 1).

**Table 1:** Treatments used in set-1 and set-2.

**Sets**	**Treatments**	**Pb (mg kg^−1^)**	**Cd (mg kg^−1^)**	**EDTA (mmol kg^−1^)**
Set-1	T0	—	—	—
	T1	1,000	—	—
	T2	2,000	—	—
	T3	2,000	—	5
	T4	1,000	200	5
				
Set-2	T0	—	—	—
	T1	—	200	—
	T2	—	400	—
	T3	—	400	5
	T4	1,000	250	5

### Morphological attributes

**Figure 1:**
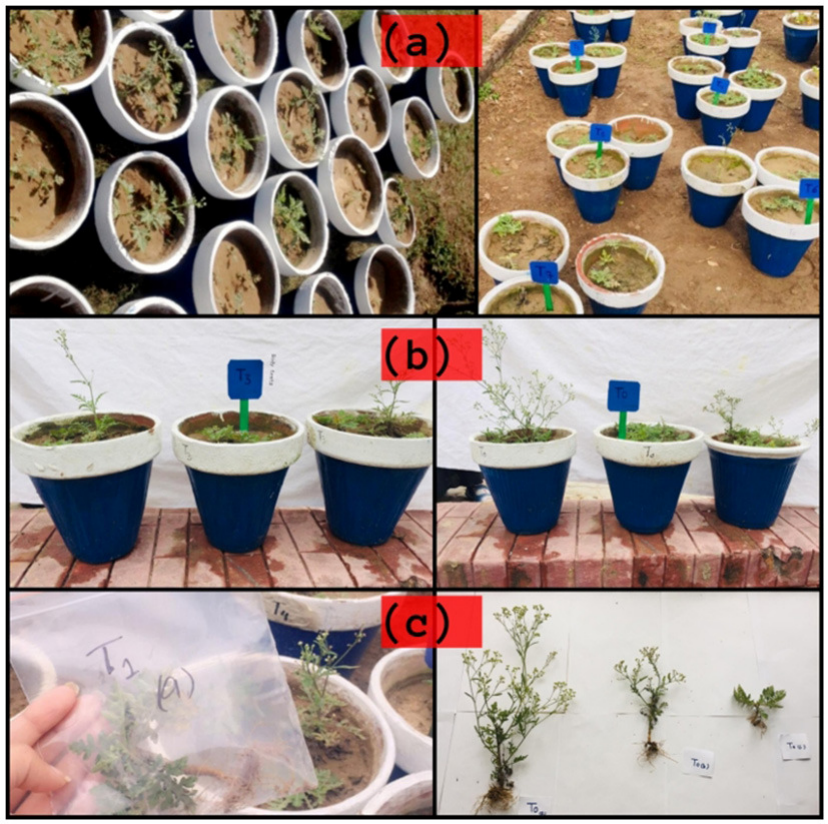
**(a)** Plants at seedling stage, **(b)** post treatment growth of seedlings, and **(c)** collection of plants for further processing.

Morphological parameters were studied in the laboratory after the collection of plant samples three months after the application of treatments. Their visuals attributes are given in Figure 1. Each plant sample was measured in terms of its height (cm), shoot length (cm), and root length (cm) with the help of a meter rod. Using digital balance, root and shoot fresh weights were measured in grams and the data were recorded. For dry weights, firstly, samples of shoots and roots have been dried for three days at 72°C in an oven. The dry weights of these samples were measured. Similarly, the total number of leaves in each pot was measured and the leaf area in each pot was measured following below formula:


$$\begin{array}{l} \text{Leaf area}=\;\left(\text{Length}\times \text{Width}/\text{Total no}.\text{of leaves}\right). \end{array}$$

### Determination of photosynthetic pigments

The method of Arnon (39) was followed for the determination of photosynthetic pigments. Leaf samples were collected separately from each pot in triplicates. In total 0.2 g of fresh leaves of each sample were taken and ground well separately. Then the grounded samples were mixed up with 80% of acetone. Ten milliliter of each plant triplicates were made by mixing 80% of acetone and were placed in a dark place in the laboratory for 48 hours. Then the samples were run on a centrifuge machine to collect the supernatant which was then analyzed in a spectrophotometer (Hitachi, Model U2001, Tokyo, Japan). The absorbance of solution was measured at 480, 645, and 663 nm for carotenoids, chlorophyll a, and chlorophyll b, respectively. The following formulae were used:


$$\begin{array}{lll} \text{Carotenoids }\left(\text{mg}/\text{g f}.\text{wt}.\right) & = & \;\left[\text{Acar}/\text{EM}\right]\;\times \text{1}000 \end{array}$$

$$\begin{array}{l} \text{Total Chl}.(\text{mg/gf}.\text{wt}.)\text{ = }[\text{20}.\text{2}(\text{O}.\text{D}.\text{ 645})\text{ − 8}.\text{02}(\text{O}.\text{D}.\text{ 663})]\;\\ \;\times V\text{/1000}\times W\;\\ \text{ Chl}.\text{a}(\text{mg/gf}.\text{wt}.)\text{ = }[\text{12}.\text{7}(\text{O}.\text{D}.\text{ 663})\text{ − 2}.\text{69}(\text{O}.\text{D}.\text{ 645})]\;\\ \;\times V\text{/1000}\times W\;\\ \text{ Chl}.\text{b}(\text{mg/gf}.\text{wt}.)\text{ = }[\text{22}.\text{9}(\text{O}.\text{D}.\text{ 645})\text{ − 4}.\text{68}(\text{O}.\text{D}.\text{ 663})]\;\\ \;\times V\text{/1000}\times W\\ \text{ Acar = O}.\text{D}.\text{ 480 + 0}.\text{114}(\text{O}.\text{D}.\text{ 663})\;\\ \text{ − 0}.\text{638}(\text{O}.\text{D}.\text{ 645})\;\\ \text{ V = Volume of the sample }\\ \text{ EM = 2500 }\\ \text{ V = Volume of the sample}. \end{array}$$

### Determination of heavy metals and ions

Heavy metals (Cd and Pb) and ions such as Na, K, and Ca concentrations in root and shoot samples were determined using the following procedure. Plant samples were collected and dried in an oven for three days at 72 °C, and then the dried-out material was ground into a powder with a pestle and mortar. For acid digestion, 0.2 g of dried material was taken in 100 ml sized beakers, and 20 ml of pure nitric acid was added and heated to 250°C on a hot plate. These beakers were removed from the hot plate and cooled down, when 10 ml of the solution remained then 10 ml of perchloric acid was added to these beakers and again heated on the hot plate until the contents became clear and fumes stopped evolving completely. Beakers were cooled down and filtered using filter papers (Whatman No. 2) and poured the solutions into cleaned sample bottles separately. Distilled water was added to each sample to make up a 100 ml solution. This extract was used for the determination of heavy metals and ions (40). Following acid digestion of the extracted samples, Atomic Absorption Spectrophotometer (Model: Perkin Elmer Analyst 100) was used to measure Na, Cd, and Pb concentrations, whereas flame photometer was used to measure Na and K concentrations (Model: Flame photometer 410, CORNING).

### Translocation factor

A plant's ability to translocate heavy metals from roots to shoots and leaves was measured by its translocation factor (TF). Meanwhile, the shoot/root bioconcentration factors, translocation efficiency, and removal efficiency were calculated using these formulas (41, 42).

1. TF = the ratio of metal concentration in the shoot/metal concentration in the root.

### Statistical analysis

The results were statistically examined using SPSS computer software's one-way analysis of variance (V 23). Non-significant was defined as a *P*-value greater than 0.05. The connection between Pb and Cd Translocation Factors in two sets of treatments was determined using a linear regression test.

## Results and discussion

### Determination of metals

**Figure 2:**
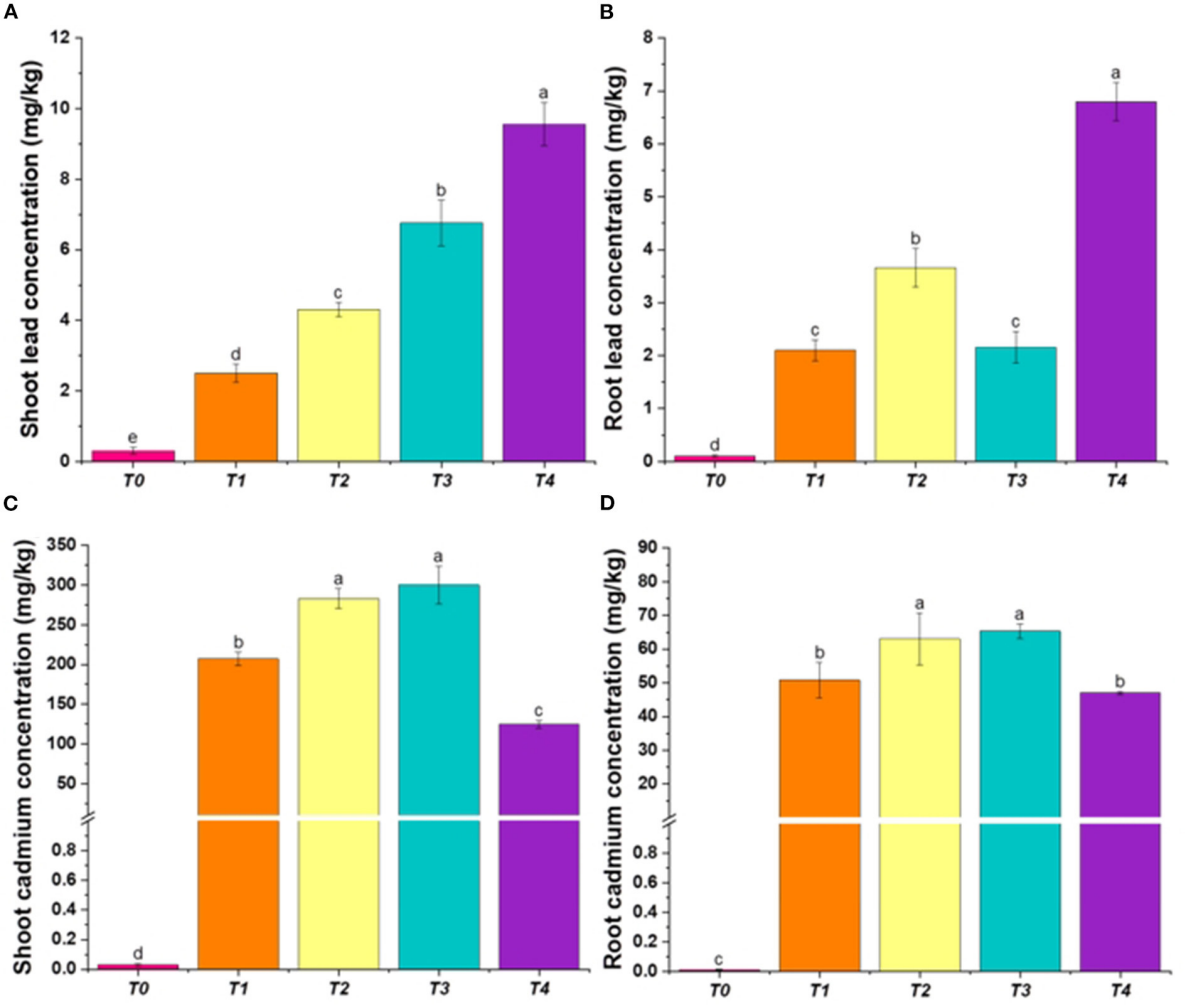
Heavy metals in *P. hysterophorus* under the effect of various treatments; **(A)** Pb concentration in *P. hysterophorus* shoot, **(B)** Pb concentration in *P. hysterophorus* root, **(C)** Cd concentration in *P. hysterophorus* shoot, and **(D)** Cd concentration in *P. hysterophorus* root.

*P. hysterophorus* accumulated higher concentration of Pb and Cd (*p* < 0.001) in roots and shoots (Tables 2, 3). The pattern of Pb and Cd accumulation was greater in the shoots than in the roots in all treatments (Figure 2). The maximum concentration of Pb was recorded in T4 in shoots as well as in roots (Figures 2A,B). In the case of Cd in set-2, maximum concentration (854.0 ± 25.2) was recorded in shoots in T3 (Figures 2C,D). However, T2 and T3 of set-2 show slight difference to each other, they differed highly significantly from the T0 though. In treatments T3 and T4, the improved accumulation of Pb and Cd in set-1 and set-2 respectively could be attributed to the addition of EDTA in these metal treatments. Our findings are consistent with prior studies, which indicated that EDTA had a considerable influence on the accumulation of Cd and Pb in plants (43, 44). A past study according to our findings reported that when the concentration of 0.25 mM EDTA was increased, fast absorption of Pb occurred in the shoot (45). One dosage of 10 mmol EDTA kg^−1^ enhanced. Ni, Cd, and Pb uptake in *Brassica rapa* and also increased their TF as there was a significantly larger concentration of these metals in upper plant parts as compared to the non-treated ones (46). The chelate-assisted phytoextraction technique appears to be more effective than a strategy for cleaning up Pb-contaminated soils that relies on the natural potential of some wild plant species (47, 48), these results have shown positive correlation with our study. The metal absorption with EDTA was also consistent with the findings of Madrid et al. (49), who found that EDTA was particularly effective at mobilizing metals from soil to the plant and can promote root-to-shoot translocation. Similarly, Turgot discovered in an investigation that 0.1 g/kg EDTA boosted total shoot: root translocation (50). According to (51), the addition of EDTA at rates of 2.5 or 5.0 mmol kg^−1^ considerably raised metal concentrations in plant shoots. High biomass plants may be useful for phytoextraction of heavy metals when exposed to large concentrations of chelate-solubilized materials (51). In calcareous soils, EDTA gradually increases the mobility of Cd and Pb (52). EDTA-enhanced metal absorption by plant roots has already been documented in several prior studies (53–55).

**Table 2:** ANOVA of various attributes of *P. hysterophorus* in response to Set-1 of treatments.

**Source of variance**	**df**	**Root length**	**Shoot length**	**Root FW**	**Shoot FW**	**Root DW**	**Shoot DW**	**No. of leaves**	**Leaf area**	**Chlorophyll a**	**Chlorophyll b**	**Total chlorophylls**	**Carotenoids**	**Calcium (roots)**	**Calcium (shoots)**	**Potassium (roots)**	**Potassium (shoots)**	**Sodium (roots)**	**Sodium (shoots)**	**Pb (roots)**	**Pb (shoots)**
**Treatments**	4	20.88**	234.4***	0.684**	37.97***	0.076***	0.602***	143.4***	0.033ns	0.184ns	0.043*	0.404ns	0.479***	12.15 ***	20.88***	5631***	3.721***	1256***	1720***	18.59***	39.23***
**Error**	10	3.030	11.82	0.110	1.425	3.968	0.001	9.466	0.010	0.081	0.011	0.149	0.034	0.064	0.034	4940	3546	1020	4666	0.083	0.183

^***^, *P* < 0.001; ^**^, *P* < 0.01; ^*^, *P* < 0.05; ns, *P*>0.05.

**Table 3:** ANOVA of various attributes of *P. hysterophorus* in response to Set-2 of treatments.

**Source of variance**	**df**	**Root length**	**Shoot length**	**Root FW**	**Shoot FW**	**Root DW**	**Shoot DW**	**No. of leaves**	**Leaf area**	**Chlorophyll a**	**Chlorophyll b**	**Total chlorophylls**	**Carotenoids**	**Calcium (roots)**	**Calcium (shoots)**	**Potassium (roots)**	**Potassium (shoots)**	**Sodium (roots)**	**Sodium (shoots)**	**Cd (roots)**	**Cd in (shoots)**
**Treatments**	4	19.49**	237.6***	0.851**	45.32***	0.082***	0.607***	148.5***	0.048**	0.369***	0.078**	0.749***	0.624***	27.59***	111.3***	2.676***	8208***	1256***	14913***	2190.0***	3276***
**Error**	10	2.250	1.866	0.077	1.047	2.163	0.005	9.533	0.004	0.028	0.011	0.058	0.029	1.305	0.0513	3686	9133	1020	42000	14.63	432.9

^***^, *P* < 0.001; ^**^, *P* < 0.01; ^*^, *P* < 0.05; ns, *P*>0.05.

### Determination of ions

**Figure 3:**
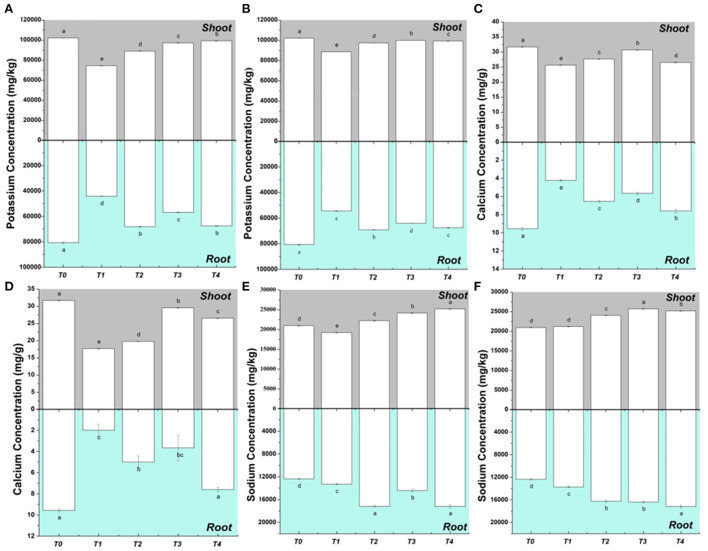
Mineral ions (K, Ca, and Na) contents in *P. hysterophorus* under the effect of various treatments in two sets. **(A)** K concentration in *P. hysterophorus* in set-1, **(B)** K concentration in *P. hysterophorus* in set-2, **(C)** Ca concentration in *P. hysterophorus* in set-1, **(D)** Ca concentration in *P. hysterophorus* in set-2, **(E)** Na concentration in *P. hysterophorus* in set-1, and **(F)** Na concentration in *P. hysterophorus* in set-2.

The ionic concentrations of K, Na, and Ca increased in roots and shoots with EDTA as compared to without EDTA treatments other than the control in both sets. In comparison to the control, T1 had the greatest fall in K level in both roots and shoots (Figures 3A,B). An increase in K in T3 and T4 compared to T1 might be related to EDTA in these treatments. In the case of Ca, T3 and T4 (with EDTA) showed maximum concentration whereas T1 and T2 (without EDTA) showed less concentration of Ca in the roots and shoots of both sets (Figures 3C,D). Nutrient ions take a vital role in cell turgor, plant development, and metabolism. Generally, lower growth rates in plants are caused by the deficiency of K inside the cells. The cytosolic roles played by the K cannot be fulfilled by other cations; hence a certain portion of plant biomass contains K (56). Ca ions are taken in by plants *via* non-selective channels in the cell membranes of their root systems. These non-specific channels also permit other divalent and some monovalent ions to pass through them (57). Ca is an important cell signaling component and helps the plants to get over various stresses such as temperature shock, changes in nutrient status, mechanical stimuli, pathogen attack, and drought (58). A lot of studies have reported the reduction in the concentration of K and Ca ions with increased concentration of Pb and Cd in the environment (59, 60). However, some studies, in accordance with our results, have reported an increase in plant K and Ca ions in the application of EDTA as compared to treatments where EDTA was not given (61).

The concentration of Na increased in both shoots and roots after applying treatments (Figures 3E,F). Na concentration in roots and shoots was found to be maximum in T4 when compared to the T0. Thus, the results showed that in the presence of the EDTA plant ionic concentration increases as compared to the treatments where EDTA was not applied. Pb and Cd concentrations have reportedly been linked to an increase in Na concentration in *P. hysterophorus*.

**Figure 4:**
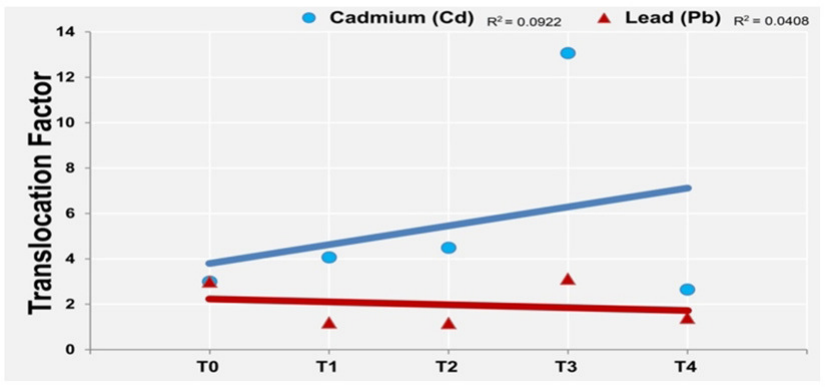
Linear correlation between translocation factors of Pb and Cd in *P. hysterophorus*.

We determined considerably high values of TF for both metals in both sets of treatments (Figure 4) which shows the extremely high capacity of *P. hysterophorus* to translocate these metals from roots to shoots. We could not find a significant correlation between TFs of *P. hysterophorus* for Pb and Cd. But generally, the TF of *P. hysterophorus* for Cd was greater than Pb. Sorghum and alfalfa have been reported to extract the heavy metal, and transfer them to the upper part and the value of translocation factor increases as the samples as treated with EDTA (62).

### Morphological attributes

**Figure 5:**
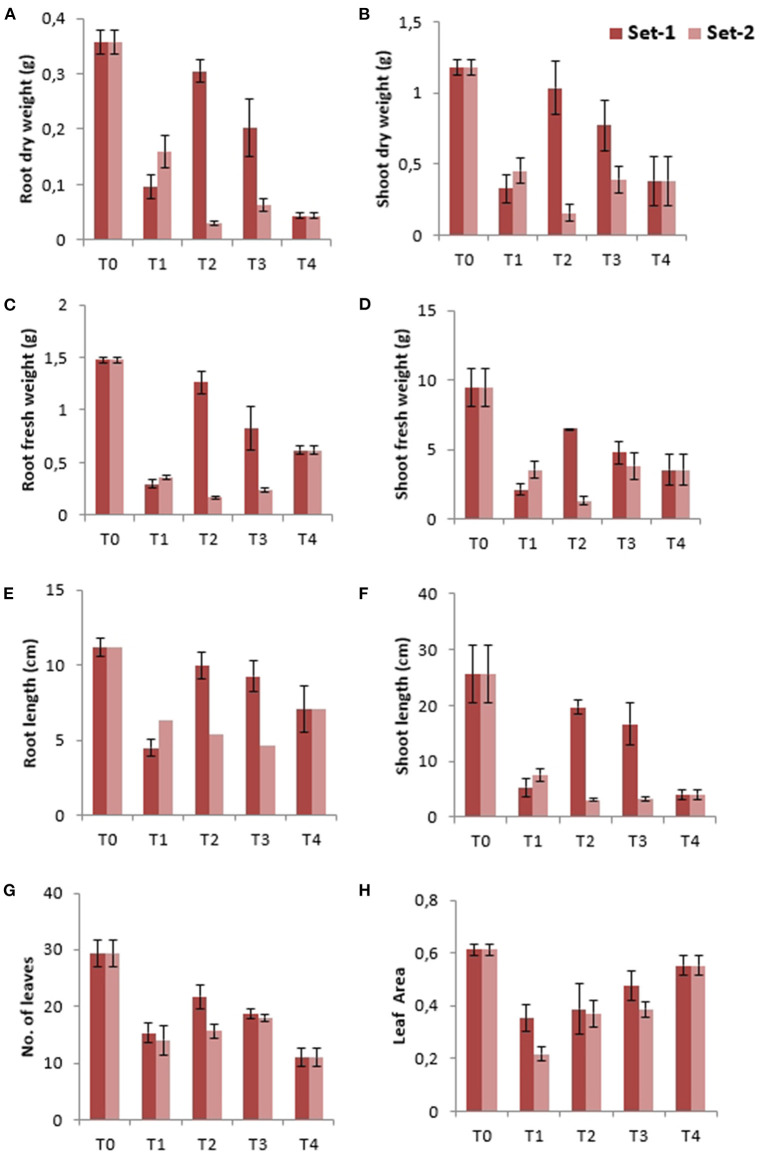
Morphological attributes of *P. hysterophorus* in two sets of treatments. **(A)** root dry weight, **(B)** shoot dry weight, **(C)** root fresh weight, **(D)** shoot fresh weight, **(E)** root length, **(F)** shoot length, **(G)** number of leaves, and **(H)** leaf area.

The fresh and dry weight of shoots and roots decreased significantly (*P* < 0.001) after the application of treatments in both sets (Figures 5A,B and Tables 2, 3). In set-1, a minimal difference in fresh weight of shoots and roots was found in T2 when compared with the control (Figures 5C,D). Shoot and root lengths of *P. hysterophorous* were also found to be affected, minimum and maximum reductions in shoot lengths of set-1 and set-2 were found in T2, respectively (Figures 5E,F). However, in set-2, T3 had the greatest reduction in root length, followed by T2. The height of the plant got decreased and showed a stunted appearance. The lowest number of leaves and maximum leaf area of the plant were seen in T4 compared to all the treatments other than the control in both sets (Figures 5G,H). EDTA seemed to have a positive impact on shoot/root fresh and dry weights in T3 and T4 compared to T2 in set-2 in accordance with the results obtained by Kanwal et al. (63). Sudan grass and sweet sorghum grow and produce more biomass when EDTA is used as a treatment in soil (64, 65). The (EDTA + Cd) combined treatments applied to *P.hysterophorous* have significantly increased plant growth and biomass, these results also showed a positive correlation with Hayat et al. (66). EDTA treatment boosted plant growth, yield, chlorophyll content, gas exchange properties and photosynthetic parameters, by increasing antioxidant enzyme activity, and it also increased metal absorption in *Brassica napus*. (67). Another study also showed that sorghum plants grew more quickly and absorbed more nutrients when treated with metal-EDTA chelate (68). Chen et al. (69) also reported higher soybean leaf mass in the presence of EDTA under Cd stress condition.

Past research has also reported that plants exposed to Pb and Cd toxins have reduced root and shoot growth, as well as lower weight (11, 12). The increasing amount of Pb in plants is shown to cause visual phytotoxic effects on plants such as chlorosis and necrosis, which results in a reduced number of, leaves (70). Reduced root weight of soybean and dry biomass in Parthenium were noted as a result of Cd toxicity (44, 69). Reduced plant growth is a common symptom of metal toxicity. Heavy metal uptake happens in plants through the ion transporters in cell membranes that are meant to transport nutrient ions thereby restricting the normal metabolic activities of the plants and hampering their growth (59, 71).

### Physiological attributes

**Figure 6:**
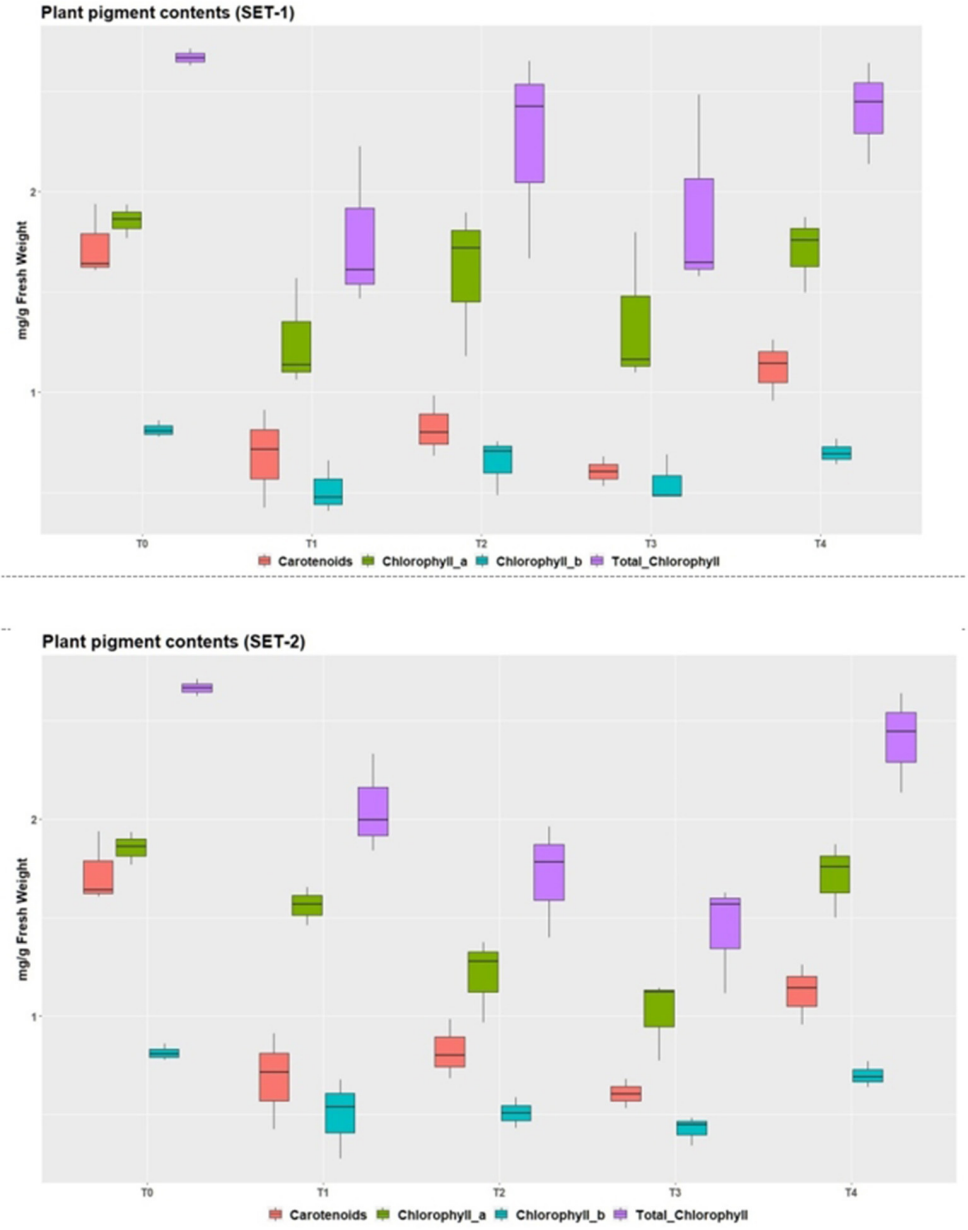
Chlorophyll pigments in *P. hysterophorus* under the effect of various treatments in two sets.

Chlorophyll a, chlorophyll b, total chlorophyll content, and carotenoids all decreased as metal intake increased. The effect of different treatments on chlorophyll contents is highly significant in both sets (Tables 2, 3). Chlorophyll a and b showed a decreasing trend from T1 to T3, but then their increase in T4 showed a positive impact EDTA had in this treatment along with metal in both sets (Figure 6). Similarly, carotenoids also showed the same trend in both sets. Similar results were obtained by other researchers as well (63, 72–75). Results are also in accordance with the Hayat et al. (66) stated that EDTA treatment raised chlorophyll content and improved plant physiology. EDTA in plants considerably enhanced plant chlorophyll content and gas exchange properties (67). The application of EDTA considerably increased the levels of chlorophyll a, b, total chlorophyll, and carotenoid content in the leaves of *B. Napus* L (63).

Chlorophyll contents are the most important biomolecules in plant cells as photosynthesis and productivity depend upon their concentration (76) noticed the direct and significant effect of heavy metals uptake on proline synthesis while a 4% reduction in chlorophyll content was seen in all the resistant/indicator species of heavy metal polluted regions. Heavy metals reduce chlorophyll contents in plants in the vicinity of leather industries of Sialkot, Pakistan (1). Various studies have shown that Cd and Pb have deleterious effects on plant chlorophyll concentration which ultimately lead to reduced photosynthetic rate and strength of the plants (5, 61, 77–79).

## Conclusions

It is concluded that the Cd and Pb concentrations in shoots and roots of *P. hysterophorus* were substantially high. The capacity of *P. hysterophorus* to accumulate Pb and Cd was shown to be increased with the addition of EDTA to the soil. Morphological attributes of *P. hysterophorus* such as shoot/root length, fresh and dry weights, leaf count, and leaf area were adversely impacted by the toxic effects of Pb and Cd, however, EDTA was found to be having a positive impact by helping *P. hysterophorus* in overcoming the negative effects of heavy metals. Similarly, the same trend was recorded for photosynthetic pigments. Ions i.e. K and Ca got reduced in Pb and Cd treatments, But EDTA in T3 and T4 helped in restoring the amount of these ions in *P. hysterophorus*. Na concentration, however, in all of the treatments except the control, was found to be significantly higher. We conclude that *P. hysterophorus* can uptake and accumulate high concentrations of Cd and Pb and this ability can be enhanced by the application of EDTA in the soil. We recommend using this highly proliferating plant species for remediation of Pb and Cd contaminated soils, but further research is required in this regard in the natural environment and for some other metal types. Decreasing the toxic level of heavy metals in the soil in accordance to the WHO standards can improve the ecosystem as well as general public health that is the prime objectives of scientists.

## Data availability statement

The original contributions presented in the study are included in the article/supplementary material, further inquiries can be directed to the corresponding authors.

## Author contributions

UE: conceptualization, data curation, formal analysis, investigation, methodology, software, validation, visualization, and roles/writing—original draft. SK: conceptualization, formal analysis, funding acquisition, investigation, methodology, project administration, software, supervision, validation, visualization, and writing—review and editing. MA: software, supervision, validation, visualization, and writing—review and editing. NK: supervision, methodology, resources, validation, visualization, and roles/writing—editing original draft. WS: formal analysis, investigation, methodology, software, validation, and visualization. NN: formal analysis, investigation, methodology, validation, and visualization. AR: visualization, supervision, project administration, funding acquisition, and writing—review and editing. HH, JY, and GY: project administration and funding acquisition. All authors contributed to the article and approved the submitted version.

## Conflict of interest

The authors declare that the research was conducted in the absence of any commercial or financial relationships that could be construed as a potential conflict of interest.

## Publisher's note

All claims expressed in this article are solely those of the authors and do not necessarily represent those of their affiliated organizations, or those of the publisher, the editors and the reviewers. Any product that may be evaluated in this article, or claim that may be made by its manufacturer, is not guaranteed or endorsed by the publisher.
